# Application of a Saddle-Type Eddy Current Sensor in Steel Ball Surface-Defect Inspection

**DOI:** 10.3390/s17122814

**Published:** 2017-12-05

**Authors:** Huayu Zhang, Mingming Zhong, Fengqin Xie, Maoyong Cao

**Affiliations:** 1 College of Mechanical and electronic Engineering, Shandong University of Science and Technology, Qingdao 266590, China; zhanghuayu@sdust.edu.cn (H.Z.); Zhong_mm0820@sdust.edu.cn (M.Z.); 2 College of Transportation, Shandong University of Science and Technology, Qingdao 266590, China; 3 College of Electrical Engineering and Automation, Shandong University of Science and Technology, Qingdao 266590, China

**Keywords:** steel ball, saddle-type eddy current sensor, defects

## Abstract

Steel ball surface-defect inspection was performed by using a new saddle-type eddy current sensor (SECS), which included a saddle coil and a signal conditioning circuit. The saddle coil was directly wound on the steel ball’s outer bracket in a semi-circumferential direction. Driven by a friction wheel, the test steel ball rotated in a one-dimensional direction, such that the steel ball surface was fully scanned by the SECS. There were two purposes for using the SECS in the steel ball inspection system: one was to reduce the complexity of the unfolding wheel of the surface deployment mechanism, and the other was to reduce the difficulty of sensor processing and installation. Experiments were carried out on bearing steel balls in diameter of 8 mm with three types of representative and typical defects by using the SECS, and the results showed that the inspection system can detect surface defects as small as 0.05 mm in width and 0.1 mm in depth with high-repetition detection accuracy, and the detection efficiency of 5 pcs/s, which meet the requirement for inspecting ISO grade 10 bearing steel balls. The feasibility of detecting steel ball surface defects by SECS was verified.

## 1. Introduction

Bearings are one of the main components of mechanical equipment, which have a direct influence on equipment performance [1,2]. Steel balls, as the core component of bearing mechanisms, are easy to develop surface defects through collision, friction, or corrosion during processing and transportation. These defects will seriously affect the running accuracy, loading capacity, and service life of steel ball-based bearings. It is of great importance for the development of the bearing industry and its application fields to quickly and accurately detect and remove defective steel balls from the product line.

A steel ball surface-defect automatic inspection system mainly consists of a steel ball surface unfolding mechanism and a defect-inspection sensor. Commonly-used sensors for steel ball surface defect detection include vision sensors [3,4], eddy current sensors [5,6,7], and capacitive sensors [8].

A visual sensor had been used by Ng [3] to recognize the steel ball surface defects, using an annular light source as an illumination system to clearly display various defects on ball surfaces. However, this sensor can only be used for ball sampling detection. A real-time system for detecting surface defects of precision steel balls had been developed by Chen et al. [4] based on machine vision. The detection system was composed of a dual-lighting system, an unfolding mechanism, and inspection algorithms for real-time signal processing and defect detection. However, the unfolding mechanism and image-processing algorithm in the system were rather complicated.

An instrument consisting of several electromagnets and a ring-shaped capacitive sensor was designed by Kakimoto [8], yielding an instrument capable of detecting surface defects with diameter of 0.05 mm and depth of 0.01 mm on a 4 mm diameter steel ball. The system required operation under lapping oil, despite possessing a simple unfolding mechanism.

The AVIKO series of steel ball detection equipment, produced by SOMET in the Czech Republic [9], possessed the characteristics of good operability and popularity. Using eddy current and a laser to inspect steel ball surface, this equipment had the advantages of a compact structure and high detection efficiency and accuracy. Due to the long contact time between a steel ball and the unfolding wheel, the raceway of the unfolding wheel wore out easily and lost its original shape. Thus, it was necessary to frequently replace the expanding wheel. Unfortunately, expensive expanding wheel replacements greatly increase inspection costs.

Although a steel ball can be effectively inspected by the devices mentioned above, it was troublesome and expensive to do such work because either the sensor structure or the unfolding mechanism was complex. Using the one-dimensional unfolding wheel described by Zhang et al. [1] or the optimized design of a saddle-type eddy current sensor, an automatic steel ball inspection system was designed so that complete detection of steel ball surfaces was realized. Zhang et al. used a five-coil probe circumferential eddy current array (CECA) sensor to detect the surface quality of steel balls. Although the unfolding mechanism was simple and easy to process, the five-coil probe CECA sensor needed repeated experiments and debugging. In the current study, as the saddle-type eddy current sensor (SECS) coil was directly wound around the detecting mechanism and became one with it, the coil was easy to process.

## 2. Surface Unfolding Mechanism

**Figure 1:**
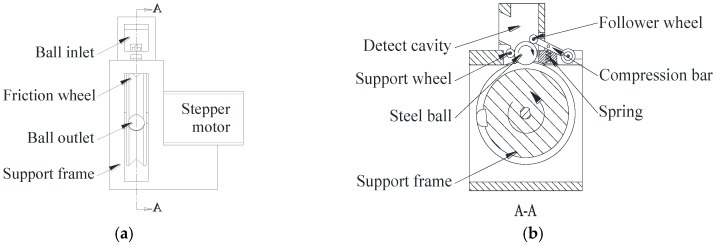

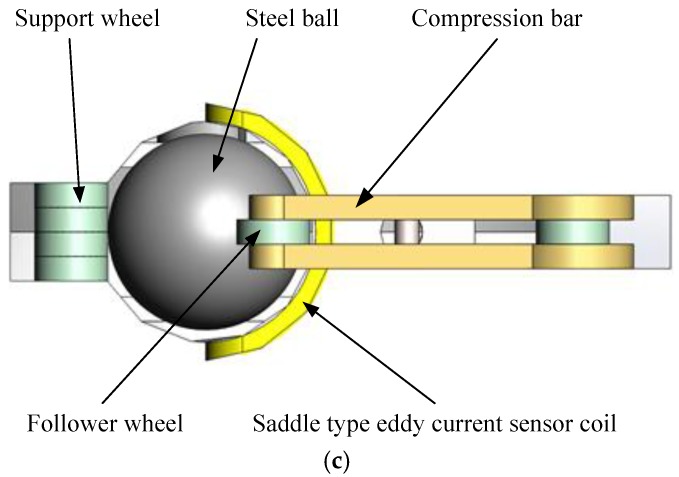
The unfolding mechanism of the steel balls. (**a**) The main view of the unfolding mechanism; (**b**) a cutaway view of the unfolding mechanism; and (**c**) the top view of unfolding mechanism.

The surface unfolding mechanism for steel balls mainly consists of a support wheel, driven wheel, friction wheel (unfolding wheel), and SECS with a saddle coil, as shown in Figure 1. Through the feeding system, steel balls to be detected regularly entered into the test chamber via the goal port. The support wheel was used to support and locate the steel ball and the driven wheel exerted a pressing force on steel ball through a pressure bar and compression spring, to prevent the ball from bouncing up and down.

The friction wheel rotated counterclockwise driven by a stepper motor via a synchronous belt, causing the steel ball to rotate in a clockwise direction, as shown in Figure 1b. When the tested steel ball rotated a full turn, surface quality information of the steel ball was fully collected and recognized by the saddle coil. With the help of the measurement circuit, this information was delivered to a computer. After comparing them with the pre-established thresholds, it could be judged whether the steel ball was defective or not.

In the process of detection, changes in the driven wheel’s supporting rod were detected by the photoelectric sensor, which indicated that a steel ball had entered into the test chamber. Then, the friction wheel started to rotate and the SECS sensor began to detect. After being completely scanned by the sensor, the steel ball left the test chamber via the ball outlet. Steel balls with defects entered into one box while defect-free balls entered into another. Concurrently, the next steel ball entered the test chamber through the inlet. Repeating the above process, automatic inspection and sorting by ball surface quality was realized.

## 3. Principles of Steel Ball Inspection with SECS

**Figure 2:**
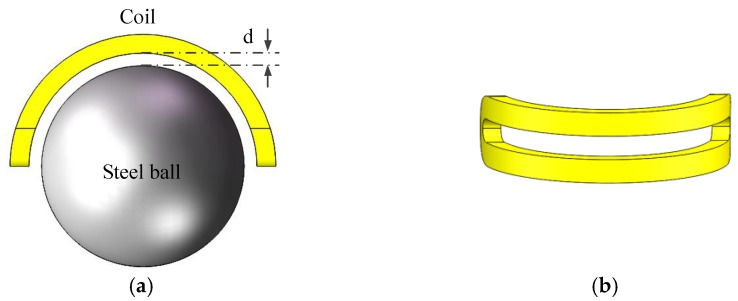
Model of steel ball surface detection based on SECS. (**a**) Distance between the coil and the steel ball; and (**b**) the structure of SECS coil.

The model of steel ball surface inspection system based on SECS is shown in Figure 2a and the SECS coil structure is shown in Figure 2b.

**Figure 3:**
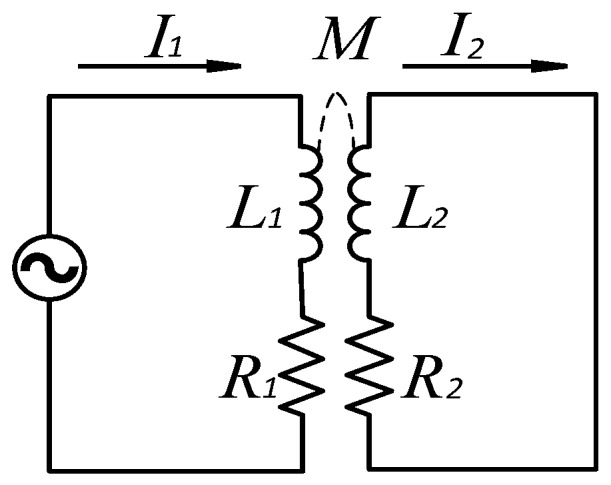
The equivalent circuit of the SECS.

According to electromagnetic induction theory, when an alternating current passes through the sensor coil, the electromagnetic alternating field, called the primary magnetic field, will be produced around it. When a steel ball moves into the magnetic field, an eddy current will be produced on the surface of it, and a secondary magnetic field created by the eddy current will be opposite to that of the sensor coil. The equivalent circuit of the eddy current sensor is shown in Figure 3, where *R*_1_ and *L*_1_ are the resistance and self-inductance of the sensor coil, which depend on the material and structure of the coil, and *R*_2_ and *L*_2_ were the equivalent resistance and self-inductance of test ball. *R*_2_ depends on the eddy current path, resistivity *ρ* of the ball; *L*_2_ depends on the eddy current path, and permeability *μ* of the steel ball [10]. The coupling degree between the two coils is characterized by the mutual inductance *M*.

According to the equivalent circuit:

(1) $$\left(R_{1}+j\omega L_{1}\right)I_{1}-j\omega MI_{2}=U\;\left(R_{2}+j\omega L_{2}\right)I_{2}-j\omega MI_{1}=0$$

where ω=2πf is the exciting angular frequency of the sensor coil, which is proportional to the frequency of current f in the SECS. M is the mutual inductance, depending on the distance between the sensor coil and the steel ball.

Therefore, the equivalent impedance of the sensor coil becomes:

(2) $$Z=(R_{1}+R_{2}\frac{\omega^{2}M^{2}}{R_{2}^{2}+\omega^{2}L_{2}^{2}})+j\left[\omega L_{1}-\omega L_{2}\frac{\omega^{2}M^{2}}{R_{2}^{2}+\omega^{2}L_{2}^{2}}\right]$$

The equivalent loss resistance *R* of the sensor coil is:

(3) $$R=R_{1}+R_{2}\frac{\omega^{2}M^{2}}{R_{2}^{2}+\omega^{2}L_{2}^{2}}$$

The equivalent inductance *L* of the sensor coil is:

(4) $$L=j\left[\omega L_{1}-\omega L_{2}\frac{\omega^{2}M^{2}}{R_{2}^{2}+\omega^{2}L_{2}^{2}}\right]$$

The *Q*-factor of the sensor coil is described as:

(5) $$Q=\frac{\omega L}{R}$$

According to the above equations, with the change of *d* between the sensor coil and the tested steel ball, the impedance *Z*, inductance *L*, and *Q*-factor are changed by the mutual inductance *M*. Parameters *Z*, *L*, and *Q* are derived as functions of *d*, *ρ*, *μ*, and ω, whereby:

(6) Z,L or Q=f(d,ρ,μ,ω)

If distance *d* and exciting frequency *f* remain unchanged, then the sensor output is a function of conductivity *ρ* and permeability *μ* of the steel ball. Therefore, a two-variable function is desirable, which is described as:

(7) Z,L or Q=f(ρ,μ)

During the process of nondestructive inspection of steel ball surfaces, the distance *d* and exciting frequency *f* remain constant. If there are defects on the steel ball surface, the resistivity *ρ* and magnetic permeability *μ* of the steel ball show significant differences from that of a defect-free steel ball. Equation (7) shows that any change in resistivity *ρ* and magnetic permeability *μ* of steel ball will result in the change of *Z*, *L*, and *Q* of the SECS coil. According to the measuring value of *Z*, *L* or *Q*, it is easy to judge whether the test steel ball is defective or not.

## 4. Measuring Circuit of SECS

*Z*, *L*, and *Q* mentioned above can be converted into electrical signals, such as current, voltage, frequency, and phase. Different circuits may be used for the converting, such as a frequency modulation circuit, amplitude modulation circuit, and bridge circuit.

**Figure 4:**
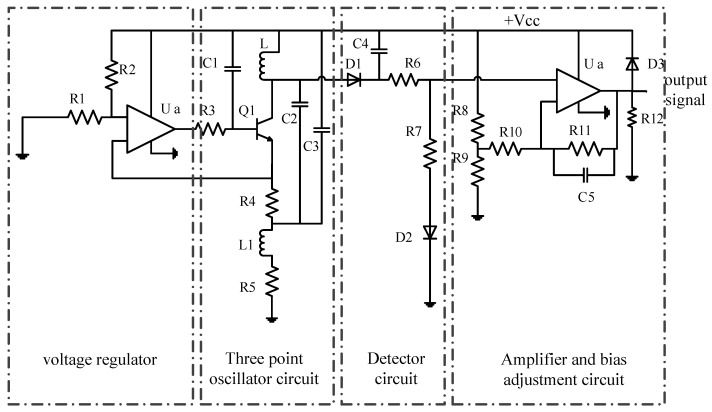
The measuring circuit of the SECS.

In this paper, a frequency modulation converting circuit was used. This is mainly composed of a three-point capacitance oscillator, an inspecting circuit, and an amplifying and biasing circuit, as shown in Figure 4. The sensor coil was connected to the oscillating circuit. The distance between sensor coil and test steel ball is kept as a constant, and if there are defects on a steel ball’s surface, the equivalent inductance value of sensor coil will change and the output voltage of oscillating circuit will change accordingly. The peak–peak value of the oscillation output voltage is extracted by an inspecting circuit. After being amplified and biased, the DC voltage output is proportionate to the equivalent inductance of the sensor coil.

*L* was used as the inductance of the sensor coil in the oscillation circuit. The signal generated by the oscillating circuit is a sine wave that fluctuates at an average value of *Vcc*, with the oscillation frequency described as:

(8) $$f=\frac{1}{2\pi \sqrt{L\frac{C_{2}C_{3}}{C_{2}+C_{3}}}}$$

The value of *C*_2_ and *C*_3_ was directly determined by the oscillatory condition and frequency value of the oscillation circuit. The linearity and sensitivity of the whole circuit is affected by the frequency value directly.

In the voltage regulation circuit, the ratio of *R*_1_ to *R*_2_ is directly affected the peak–peak value of the oscillation circuit output voltage. If the ratio is too large or too small, the sensitivity of the sensor will be seriously affected, thus, the ratio should be adjusted to a suitable value. If the output voltage is too high, saturation will occur in *Q*_1_. Conversely, if the output voltage is too low, the gradient of the output voltage will be affected, which will lead to the reduction of the sensor’s sensitivity.

Amplifying and biasing circuits were used to limit the zero point output value and to adjust the magnification of the output voltage of the sensor detection signal. Adjusted of the resistance values of resistors *R*_8_, R_9_, *R*_10_, and *R*_11_, respectively, when the distance between sensor coil and steel ball varied from 0.4 mm to 0.9 mm, the output voltage changed from 4 V to 9 V, accordingly, with a linear midpoint of 5.25 V, and then the sensitivity of the SECS was up to 10 V/mm.

## 5. Finite Element Analyses

According to Zhang et al., the *Q*-factor, the uniformity of the eddy current intensity, and the eddy current distribution were compared through the analysis of three-, four-, five-, and six-coil CECA sensors [1]. Considering the actual installation and processing difficulty of the sensor, the five-coil probe was selected as the CECA sensor.

**Figure 5:**
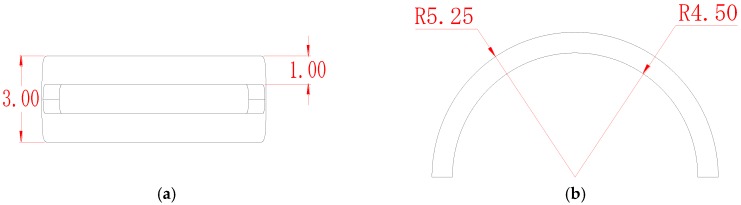
Outline dimensions of the SECS coil (the unit is mm). (**a**) The top view of coil; and (**b**) the main view of coil.

In this paper, the same unfolding mechanism was used as by Zhang et al. [1]. Parameters of the SECS coil are shown in Table 1 and the outline dimensions in Figure 5. According to the hardware circuit, the excitation frequency of the eddy current is 1 MHz, the excitation current is 0.5 A, and the coil center coincides with a steel ball’s center. In the analysis of finite elements, steel balls of G10 grade [2] were used in these electromagnetic simulations.

**Figure 6:**
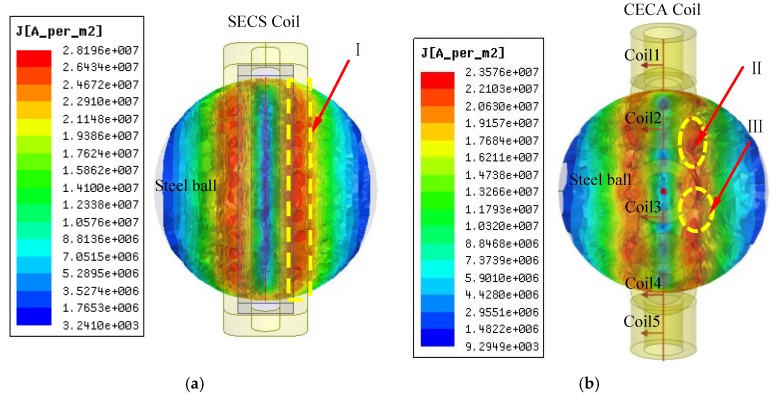
Eddy current density distribution on a steel ball surface. (**a**,**b**) Eddy current density distributions on a steel ball surface induced by SECS and by the five-coil CECA sensor, respectively.

The eddy current induced by a SECS and a CECA sensor on steel ball surfaces are shown in Figure 6a,b, respectively. In addition, the maximum eddy current density induced by the SECS on a ball is 2.8196 × 10^7^ A/m^2^ and the maximum eddy current density induced by the five-coil CECA sensor is 2.3576 × 10^7^ A/m^2^. The eddy current induced on these surfaces by the SECS is clearly more intensive than that of the five-coil CECA sensor.

In Figure 6a, the eddy current density induced by the SECS on regionⅠis 2.8196 × 10^7^ A/m^2^, which is uniform for the steel ball inspection. While in Figure 6b, the eddy current density induced by the five-coil CECA sensor is 2.3576 × 10^7^ A/m^2^ on regionⅡand 2.0630 × 107^7^ A/m^2^ on region III. The eddy current distribution by the SECS is clearly more uniform and intensive than that of the five-coil CECA sensor.

The five-coil probe CECA sensor from Zhang et al. is difficult to process and need many adjustments and debugging after being installed on the inspection mechanism [1], while the SECS coil is directly wound around the detecting mechanism and integrate with it into a whole, which greatly reduces the difficulty of sensor installation and debugging.

## 6. Experiments

### 6.1. Experimental System

**Figure 7:**
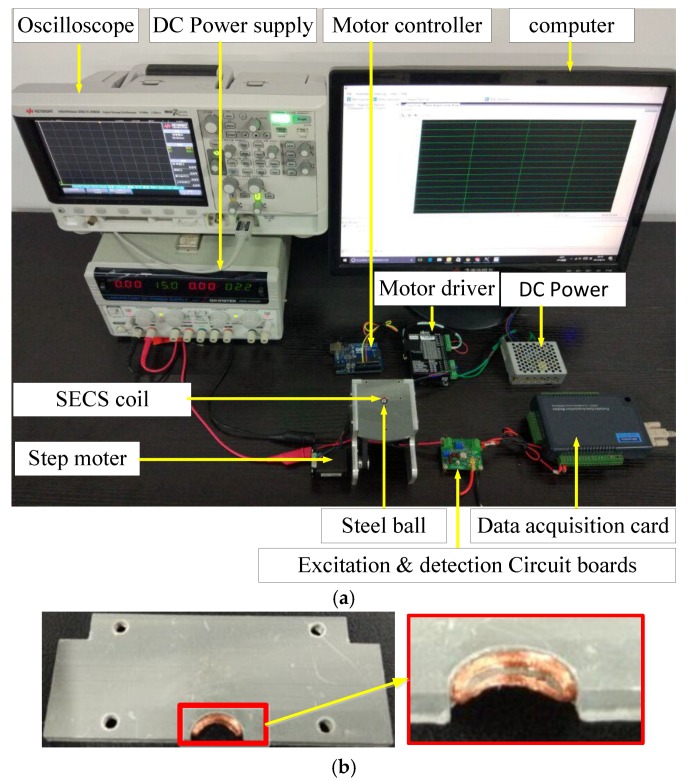
A test of the steel ball surface quality detection system. (**a**) SECS experimental testing system and (**b**) SECS coil projected and built in house.

The experimental setup of steel ball surface quality detection system is shown in Figure 7. In the detection system, the friction wheel is driven by a stepper motor, and the steel ball rotates in a one-dimensional meridional direction under the drive of the friction wheel, as shown in Figure 7a. The saddle-shaped coil is fixed on the support frame with a latitudinal direction and becomes one with the detection mechanism. The shape of the SECS coil is shown in Figure 7b. Steel balls composed of GCr15 and 8 mm in diameter were selected for the experiment. The distance between the sensor and test steel ball was set to 0.5 mm, and the exciting frequency was 1 MHz.

The bearings steel ball dropped into detect cavity one by one from the feeder. Steel ball surface quality information is collected and recognized by the saddle coil. With the help of the excitation and detection circuit, the sensor coil detection signal is amplified and converted into an electric signal with a varying voltage from 4 V to 9 V. The detection signal is Collected by a USB4711A (Advantech board, Advantech Co., Ltd., Taipei, Taiwan), and the detection signal is compared with a setting threshold, to determine whether the currently tested steel ball was defective or not. According to surface quality information from a tested steel ball, the control sorting system then performed the corresponding action, such that automatic sorting can be realized.

### 6.2. Experimental Results

**Figure 8:**
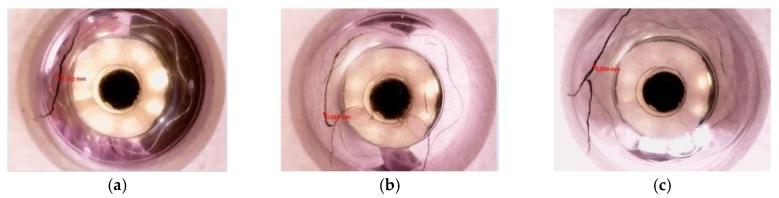
Samples of defective steel balls. (**a**) Linear cracks; (**b**) arc cracks; and (**c**) cross cracks.

Typical defects on steel ball surfaces include linear, arc, and cross cracks, as shown in Figure 8. Linear crack widths were ~0.12 mm and were ~0.5 mm in depth, arc crack had widths of ~0.05 mm and were ~0.1 mm in depth, and cross crack had widths ~0.06 mm and were ~0.9 mm in depth.

**Figure 9:**
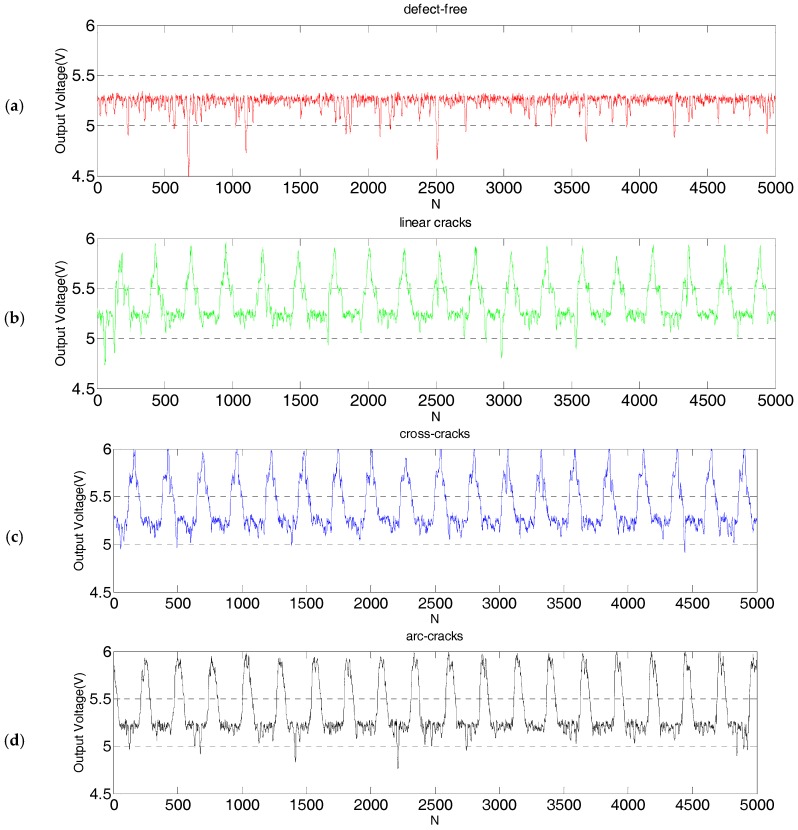
The inspection results of continuous sampling of 5000 points on a steel ball surface with a USB4711A (Advantech board, Advantech Co., Ltd., Taipei, Taiwan). (**a**) Inspection curve of steel balls without defect; and (**b**–**d**) inspection curves of steel balls with linear cracks, cross cracks, and arc cracks, respectively.

One defect-free steel ball and three steel balls with three typical defects, respectively, mentioned above were placed into the inspection system. The repeating inspection accuracy of SECS was verified by using a friction wheel without a ball outlet, and the rotation speed of the stepper motor was set to 300 r/m. These four steel balls were detected repeatedly 19 times, and 5000 data points were collected for each steel ball’s inspection signal, which are shown in Figure 9.

The maximum deviation of the peak voltage between linear cracks was 0.2 V, the maximum peak deviation between cross cracks was 0.2 V, and the maximum peak deviation of arc cracks was 0.1 V. Results showed that the eddy current sensor based on a saddle coil exhibited good repeatability and high accuracy. As the friction wheel turned one revolution, detection of one steel ball was completed. The speed of the stepper motor was set to 300 r/m, while the transmission ratio between the friction wheel and the stepper motor was 1:1, so the detection efficiency of the steel ball was 300 pieces per minute (pcs/m) or five pieces per second (5 pcs/s).

There are two points to be explained in the process of the experiment:

(1) After about 1 h of continuous running of the detection system, there was a temperature rise from 16 °C to 52 °C for the sensor coil. Through repeating experiments, it was proofed that the temperature variation had a small influence on the inspection signal, but had little influence on the judgment of the steel balls, so there is no discussion in this article.
(2) According to the design principle of the unfolding mechanism, the inspection efficiency of the steel balls depends on the rotational speed of the stepping motor. If the motor speed was over 320 r/m, the steel ball was prone to slip on the expansion wheel. This is not conducive to the full inspection of the steel ball. In this unfolding mechanism, the speed of the stepper motor was less than 320 r/m.

### 6.3. Threshold Setting and Discrimination of Steel Balls

**Figure 10:**
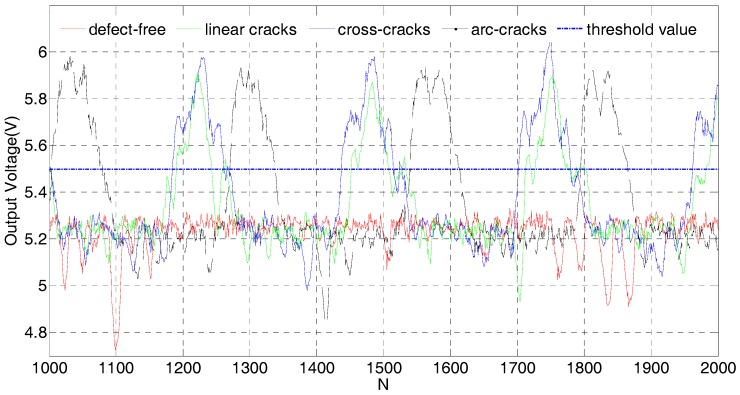
Excerpted diagram of 1000–2000 points from the 5000 points of the inspection signal. The threshold value of 5.5 V was used to distinguish whether the steel ball has a defect or not.

The waveform of the steel ball detection signal was visualized more clearly by intercepting the diagram of the detection signal from 1000 to 2000 points, as shown in Figure 10. The detection signal of the defect-free steel ball was flat with the maximum voltage amplitude of 5.3 V. There were downward excursions in some places in the detection signal; the reason for the mutation was that the machined precision of the friction wheel was inadequate, having a surface protuberance that caused a slight pulsation of the ball. At some point, the distance between the sensor coil and the steel ball was closer and the voltage amplitude was, thus, slightly reduced. For defective steel balls, the voltage amplitudes with linear cracks, arc cracks, and cross cracks were 5.85 V, 5.94 V, and 5.90 V, respectively. It is easy to see that there were clear differences between defective and defect-free steel balls. These results showed that this SECS coil could be used for the evaluation of steel ball surface quality.

In order to realize the automatic online detection of a steel ball, it was necessary to set a threshold for steel balls with the same batch. Comparing the detection value with the threshold, if the detection value was larger than the threshold, it can be concluded that the tested steel ball was defective; conversely, the steel ball was defect-free. In the experimental system, the threshold of 5.5 V was used to distinguish whether the steel ball had a defect or not.

## 7. Conclusions

A new method for detecting steel ball surface defects based on a SECS coil was proposed in this study. Uniformly-distributed eddy current density on steel ball surfaces induced by the SECS reduced the probability of missing defect detections. Experimental results showed that the repeatability of the test was good and that it could be used in a detection system of steel ball surface defects. In experiments, the speed of the friction wheel (stepper motor) was 300 r/m, with the ball surface detection at the rate of 5 pcs/s. Compared with the CECA sensor, the coil of an SECS was directly wound around the detecting mechanism, providing the advantages of simple structure and easy processing, which greatly reduced the difficulty of sensor installation and debugging.

Influence of a temperature rise on the long-running of the inspection system was not considered in the paper. In the future, we will further discuss the influence of temperature, and explore new inspection methods for improving the efficiency, such as the adoption of a novel motion-induced eddy current-based thermography (MIECT) for high-speed inspection [11].

## Figures and Tables

**Table 1:** Parameters of the sensor coil.

Parameter	Unit	Value
Coil inside radius	mm	4.50
Coil outside radius	mm	5.25
Coil thickness	mm	0.75
Distance between sensor coil and steel ball	mm	0.50
Total coil width	mm	3.00
Coil side width	mm	1.00
Enameled wire diameter	mm	0.06
Steel ball diameter	mm	8.00
Steel ball material	-	GCr15
